# Postoperative acute kidney injury in patients with gynecologic malignancies

**DOI:** 10.1186/cc13562

**Published:** 2014-03-17

**Authors:** M Didik, P Zeyneloglu, A Pirat, A Ayhan, Z Kayhan

**Affiliations:** 1 Baskent University Faculty of Medicine, Ankara, Turkey

## Introduction

Postoperative acute kidney injury (AKI) is an important cause of mortality and morbidity among surgical patients. Less is known about the occurrence of AKI after operations for gynecologic malignancies. The aim of this study was to determine the incidence of AKI in patients who underwent surgery for gynecologic malignancies and to compare patients with and without postoperative AKI.

## Methods

A total of 1,000 patients were enrolled retrospectively from January 2007 through March 2013. Patients under 18 years of age, those with chronic kidney disease and patients who died within the first week after surgery were excluded. AKI was defined according to the KDIGO 2012 Clinical Practice Guideline for Acute Kidney Injury. Perioperative variables of patients were collected from medical charts.

## Results

The mean age was 55.4 ± 12.4 years. The incidence of postoperative AKI was 8.8%, stage 1 occurred in 5.9%, stage 2 in 2.4% and stage 3 in 0.5% of the patients. Patients who had AKI were significantly older (57.9 ± 12.8 vs. 55.1 ± 12.3 years, *P *= 0.046), had higher body mass index (30.1 ± 7.1 vs. 28.6 ± 6.4, *P *= 0.031), higher preoperative C-reactive protein (CRP) levels (157.8 ± 121.5 vs. 76.6 ± 81.1 mg/dl, *P *= 0.037) and more frequently had history of distant organ metastasis (13.3 vs. 7.8%, *P *= 0.022) when compared with those who did not have AKI. When compared with patients who did not develop AKI postoperatively, longer operation times (149.1 ± 62.5 vs. 123.8 ± 56.6 minutes, *P *= 0.001), intraoperative usage of higher amounts of erythrocyte suspension (279.5 ± 616.7 vs. 128.3 ± 296.1 ml, *P *= 0.001) and fresh frozen plasma (165.9 ± 284.8 vs. 94.1 ± 185.9 ml, *P *= 0.001) were seen in those who developed AKI.

## Conclusion

Our results demonstrate that AKI occurs in 8.8% of the patients following surgery for gynecologic malignancies. Patients who had AKI were older, had higher body mass index with higher preoperative CRP levels, more frequent distant organ metastasis and longer operation times and higher amounts of blood transfused intraoperatively.

